# Whole-exome sequencing confirms implication of VPS13D as a potential cause of progressive spastic ataxia

**DOI:** 10.1186/s12883-022-02553-0

**Published:** 2022-02-12

**Authors:** Christelle M. Durand, Chloé Angelini, Vincent Michaud, Claire Delleci, Isabelle Coupry, Cyril Goizet, Aurelien Trimouille

**Affiliations:** 1 INSERM U1211, Laboratoire Maladies Rares: Génétique et Métabolisme, Bordeaux Univ., CHU Bordeaux – Hôpital Pellegrin - Place Amélie Raba Léon, 33076 Bordeaux Cedex, France; 2 Centre de Référence Neurogénétique, Service de Génétique Médicale, CHU Bordeaux, Bordeaux, France; 3 Service de Génétique Médicale, CHU Pellegrin, Bordeaux, France; 4 Service de Médecine Physique et de Réadaptation Pôle de Neurosciences Cliniques, CHU Pellegrin, Bordeaux, France; 5 Univ. Bordeaux, INSERM, BPH, U1219, F-33000, Bordeaux, France; 6 Service de Pathologie, CHU Pellegrin, Bordeaux, France

**Keywords:** Spastic ataxia, VPS13D, Whole exome sequencing, Mitochondrial network

## Abstract

**Background:**

VPS13D is a large ubiquitin-binding protein playing an essential role in mitophagy by regulating mitochondrial fission. Recently, *VPS13D* biallelic pathogenic variants have been reported in patients displaying variable neurological phenotypes, with an autosomic recessive inheritance.

The objectives of the study were to determine the genetic etiology of a patient with early onset sporadic progressive spastic ataxia, and to investigate the pathogenicity of *VPS13D* variants through functional studies on patient’s skin fibroblasts.

**Case presentation:**

We report the case of a 51-year-old patient with spastic ataxia, with an acute onset of the disease at age 7. Walking difficulties slowly worsened over time, with the use of a wheelchair since age 26. We have used trio-based whole-exome sequencing (WES) to identify genes associated with spastic ataxia. The impact of the identified variants on mitochondrial function was assessed in patient’s fibroblasts by imaging mitochondrial network and measuring level of individual OXPHOS complex subunits. Compound heterozygous variants were identified in *VPS13D*: c.946C > T, p.Arg316* and c.12416C > T, p.(Ala4139Val). Primary fibroblasts obtained from this patient revealed an altered mitochondrial morphology, and a decrease in levels of proteins from complex I, III and IV.

**Conclusions:**

Our findings confirmed implication of *VPS13D* in spastic ataxia and provided further support for mitochondrial defects in patient’s skin fibroblasts with *VPS13D* variants. This report of long-term follow up showed a slowly progressive course of the spastic paraplegia with cerebellar features. Furthermore, the performed functional studies could be used as biomarker helping diagnosis of *VPS13D*-related neurological disorders when molecular results are uneasy to interpret.

**Supplementary Information:**

The online version contains supplementary material available at 10.1186/s12883-022-02553-0.

## Background

VPS13D is a large ubiquitin-binding protein (492 kDa) which belongs to a family of 4 ubiquitously expressed genes encoding highly conserved proteins in eukaryotic cells (VPS13A–D). Recent studies have suggested that Vps13D plays a crucial role in mitochondrial dynamics and is important for mitochondrial integrity [1–3]. Indeed, in *Drosophila*, Vps13D is required for mitochondrial clearance and is essential in the mitophagy process by regulating mitochondrial fission. Moreover, Vps13D depleted neurons display an incomplete mitophagy with an accumulation of mitophagy intermediates [3]. Depletion of *VPS13D* in HeLa cells also leads to enlarged and rounded mitochondria [2]. Finally, Vps13D is localized at different membrane contact sites and may regulate mitochondria_endoplasmic reticulum contacts, as well as peroxisome biogenesis [4–7]. This localization could be largely determined by competition between specific protein adaptors, all of them binding to VAB (VPS13 adaptor binding domain) [6].

Recently, *VPS13D* pathogenic variants have been successively reported in patients displaying variable neurological phenotypes generally dominated by movement disorders including chorea, dystonia, tremor, ataxia, spastic paraplegia, spastic ataxia and seizures with highly variable age at onset [8–12]. Progressive spastic ataxia constitutes a genetically heterogeneous group of disorders characterized by simultaneous cerebellar ataxia and limb spasticity, possibly associated with other severe neurological complications [13].

We confirmed herein that spastic ataxia may be related to *VPS13D* pathogenic variants and we showed abnormal mitochondrial morphology and reduced mitochondrial OXPHOS complexes in skin fibroblasts suggesting altered mitochondrial function.

## Narrative

### Case presentation

**Fig. 1:**
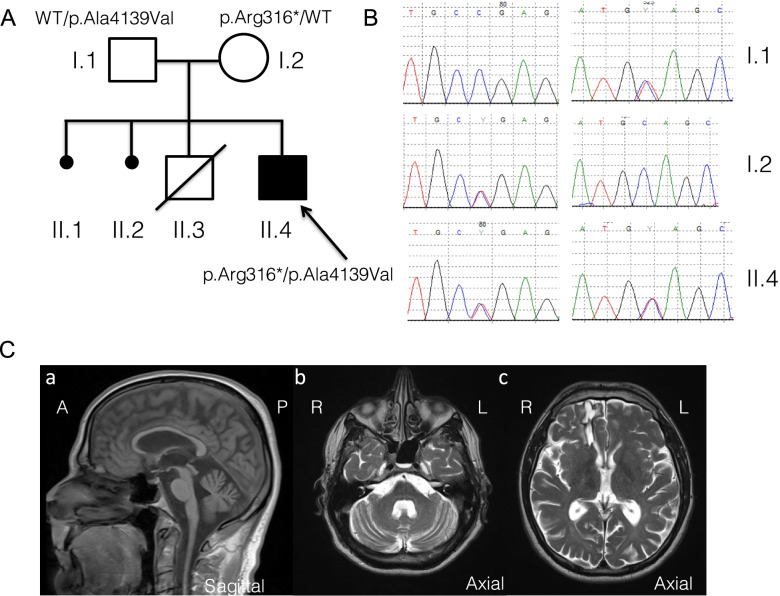
Family pedigree and brain imaging. **A** Family Pedigree. **B** WES identified two variants in *VPS13D* gene [NM_015378.3] in *trans* in patient (II.4). The first one is a nonsense variant inherited from his mother (I.2): c.946C > T, p.Arg316*; the second one is a missense variant inherited from his father (I.1): c.12416C > T, p.(Ala4139Val). WT: wild-type. **C** Brain MRI sections with (C.a) midline sagittal T1 showing vermian atrophy, (C.b) axial T2 showing pontocerebellar and cerebellar atrophy, and (C.c) axial T2, showing frontal angioma therapy after-effects. (A) Anterior; (P) Posterior; (L) Left; (R) Right

The proband (II.4) was a 51-year-old man born from unrelated healthy parents (I.1 and I.2) after an uneventful pregnancy. Familial history was negative (Fig. 1A). He had neither psychomotor delay nor behavioral disorders. His medical history began at age 7, with acute unilateral walking difficulties that progressively became bilateral. The walking difficulties were mainly due to spasticity first, with tiptoeing, with no ataxia or weakness. Walking difficulties slowly worsened over time. He walked with a stick at 19 and he’s using a wheelchair since age 26. He presented a generalized onset motor seizure at age 25 that revealed a frontal angioma, treated with radiotherapy. He presented memory impairment from age 30. Neuropsychological evaluation highlighted moderate difficulties for fronto-parietal efficiency. He complained from dysuria at age 30, and urodynamic testing showed detrusor and external sphincter dyssynergia. Clinical examination found at age 45 a tetraparesis with brisk reflexes, bilateral Hoffmann and Babinski’s sign. Spasticity was mostly found on lower limbs with atrophy of proximal muscles. Sensory function was impaired with light touch and vibration deficits, localized preferentially at the lower limbs. He also had a cerebellar kinetostatic ataxia, with truncal hypotonia. He had no extrapyramidal symptoms; cranial nerves examination was normal. No signs of dysmorphia were noted. At age 49, worsening of ataxia and swallowing troubles with episodic false passages were noted. At age 51, he presented focal onset evolving into bilateral convulsive status epilepticus revealing structural epilepsy and requiring chronic use of anti-epileptic medication. Blood analyses revealed normal levels of cholestanol, very long chain fatty acid, normal 25 and 27-hydroxycholesterol plasma level and normal organic and amino acids chromatography. No abnormality was found on urine chromatography of organic acids. At age 51, brain MRI showed atrophic cerebellum mostly on the vermis and an atrophic spinal cord. After-effects of frontal angioma were also present (Fig. 1C).

### Whole-exome sequencing (WES), analysis, and interpretation

We decided to perform trio-based whole-exome sequencing to investigate this patient. Library preparation, exome capture, sequencing and data analysis have been performed by IntegraGen SA (Evry, France), using Twist Bioscience in-solution enrichment methodology (Agilent, Santa Clara, California), followed by paired-end 75 bases massively parallel sequencing on Illumina HiSeq4000 (Illumina, San Diego, California). Whole-exome sequencing for the patient and his parents identified compound heterozygous variants in *VPS13D* gene [NM_015378.3], with appropriate familial segregation (Fig. 1A, B): c.946C > T, p.Arg316* and c.12416C > T, p.(Ala4139Val). Sanger sequencing using standard protocol was performed to confirm the presence of these variants.

The first variant (rs758368974) was a nonsense variant only present twice at heterozygous state in gnomAD database and considered as pathogenic according to the ACMG guidelines. The second variant was a missense variant absent from gnomAD and predicted as deleterious by PolyPhen-2, LRT and Mutation Taster software. It has a CADD-Phred score of 24.9. These two variants have been submitted in the Clinvar database (SCV001338779.1 and SCV001338853.1).

### Mitochondrial studies

**Fig. 2:**
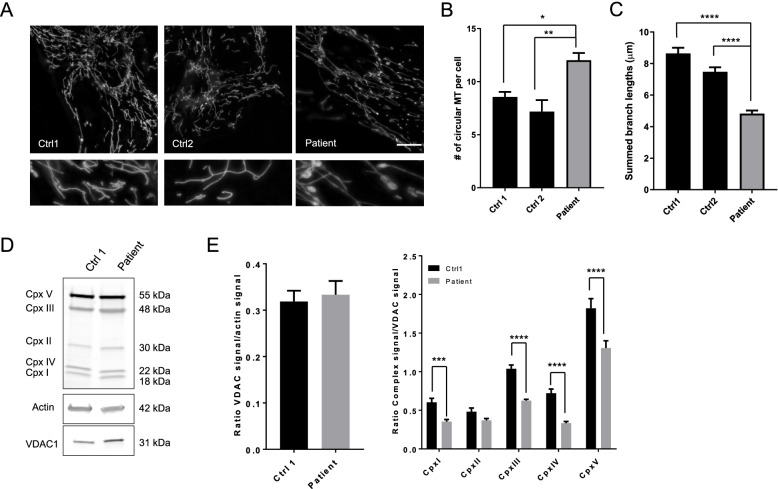
Patient skin fibroblasts display abnormal mitochondrial morphology and a reduction of mitochondrial complexes. **A** Representative images of mitochondrial network in 2 healthy subjects and patient fibroblasts stained with Green mitotracker®. 3D-Images projection were obtained using a Zeiss Vivatome microscope (objectives × 63, scale bar = 10 μm). **B** Quantification of mitochondrial morphology by calculating number of circular mitochondria (MT) per cell [8]. **C** Summed branch length were measured with Mitochondrial Network Analysis (MiNA) toolset ImageJ plug-ins [15]. Data are represented as mean ± SEM from 5 independent experiments with 80 to 100 cells. * *p* < 0.05, ** *p* < 0.01, **** *p* < 0.0001. **(D-E)** Evaluation of mitochondrial respiratory complex proteins quantity in patient fibroblasts. Western blot analyses of mitochondrial OXPHOS complex subunits in patient and control (ctrl1) fibroblasts using β-actin (Sigma) and VDAC1 (Porin, Abcam) as loading controls. Total OXPHOS Antibody Cocktail® (Abcam) contains: ATP synthase 5A (Complex V, 55 kDa), COX II (complex IV, 22 kDa), UQCRC2 (complex III, 48 kDa), SDHB (complex II, 30 kDa), and NDUFB8 (complex I, 18 kDa). Data are expressed as mean ± SEM from 8 independent experiments. Statistical analyses were performed with GraphPad Prism 7 using Mann Whitney test or 2 Way ANOVA multiple comparisons tests (GraphPad Software, Inc.)

Given the known role of VPS13D on mitochondrial network and integrity, we investigated mitochondrial function and morphology in the patient and in two healthy subjects including one age and gender matched control (ctrl1). Primary fibroblasts were obtained from arm skin biopsies and were cultured under standard conditions [14]. To study mitochondrial morphology, mitochondrial imaging was performed with MitoTracker Green (Fig. 2A). The number of circular mitochondria were quantified using ImageJ software and expressed as a percentage of circular mitochondria per cells as previously described [8]. Patient fibroblasts presented a significant increase of bright and circular mitochondrial objects compared to controls, as previously observed [8], suggesting a structural defect in mitochondria (Fig. 2B). Moreover, by using the Mitochondrial Network Analysis tool from ImageJ platform (MiNA) [15], we showed that summed branch lengths were significantly reduced in patient fibroblasts confirming an altered mitochondrial network (Fig. 2 C). To investigate the effect of *VPS13D* mutations on the steady-state levels of individual OXPHOS complex subunits in fibroblasts, western blot analysis was performed (Fig. 2) on the fibroblast lysates from patient and ctrl1. Results revealed that in patient cells, there was no apparent effect on the abundance of VDAC1, a mitochondrial outer membrane protein, suggesting no difference in mitochondria content (Fig. 2D). However, a significant decrease in steady-state levels of proteins from complex I, III and IV normalized to VDAC1 was observed in patient fibroblasts (Fig. 2E).

## Discussion and conclusions

*VPS13D*-related movement disorders have been very recently recognized and to date, only 27 individuals from 18 families have been identified [8–13]. Two main clinical presentations may be distinguished, essentially correlated with age at onset. The first phenotype occurs early after birth, in neonatal or infancy period of life before age 5 years, and leads to evident developmental delay with progressive movement disorders such as chorea, dystonia, spastic paraplegia, ataxia, spastic ataxia and seizures. The disease progressively worsens requiring assistance for walking and wheelchair in early adulthood. Brain MRI abnormalities, suggestive of Leigh syndrome, are frequent. The second phenotype occurs between late childhood and adulthood, after normal early development. The oldest previously reported age at onset was 63 years [10]. The clinical features are then essentially represented by a pure spastic paraplegia or spastic ataxia with tremor and dystonia with progressive course. In this study*,* we report a novel patient displaying *VPS13D*-related movement disorders characterized by spastic ataxia which started at age 7, which corresponds to the second phenotype described above. Cerebellar features became the core motor features around his fifties. In addition, cognitive impairment worsens progressively and is considered one of the most disabling features. However, these cognitive declines may be associated to both sequelae of a treated frontal angioma and potentially undiagnosed focal epilepsy rather than directly caused by the disease.

This patient harbors compound heterozygous nonsense and missense variants as appears to be most commonly found in VPS13D-affected patients [8–12]. Our observations confirm that *VPS13D* seems intolerant to complete loss of function of both alleles, being likely incompatible with life. Several studies in *Drosophila* and yeast have shown a major role of Vps13 in two processes that are essential to mitochondrial health: mitochondrial fission and mitophagy [1–3, 7, 8]. Our functional studies in this patient’s fibroblasts confirm that mutation in *VPS13D* induces defects in mitochondrial network and function. Our work support abnormal mitochondrial morphology previously shown in other patients [8]. Moreover, the decrease of some OXPHOS complex subunits observed here argues for additional alteration of mitochondrial function. Further investigations are required to potentially use abnormal mitochondrial morphology as a trait biomarker helping diagnosis of *VPS13D*-related neurological disorders when molecular results are uneasy to interpret and to better understand the functional mitochondrial role of the protein.

## Supplementary Information

**Additional file 1.**

## Data Availability

The datasets used and/or analyzed during the current study are available from the corresponding author upon reasonable request.

## Abbreviations

WES: Whole exome sequencing
MiNA: Mitochondrial Network Analysis toolset ImageJ plug-ins
OXPHOS: Oxidative phosphorylation
