# Supplementary material for: Whole-exome sequencing confirms implication of VPS13D as a potential cause of progressive spastic ataxia
Source: BMC Neurol. 2022 Feb 12;22:53. doi: 10.1186/s12883-022-02553-0 (PMC8840315; doi:10.1186/s12883-022-02553-0)
Supplement: Supplementary file 1 — Additional file 1. [file 12883_2022_2553_MOESM1_ESM.pptx]

## Slide 1
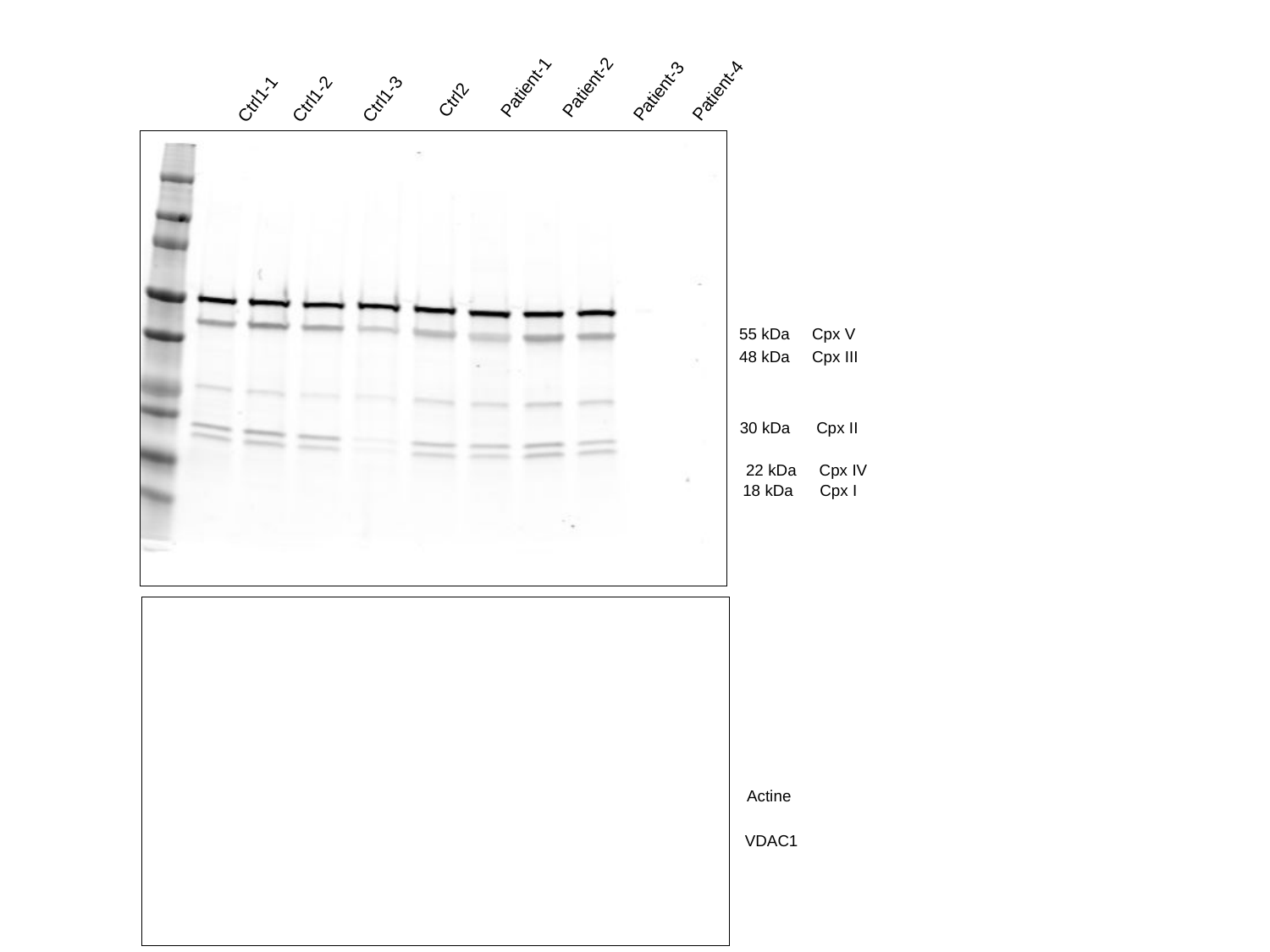

Patient-1
Patient-2
Patient-4
Patient-3
Ctrl1-1
Ctrl1-2
Ctrl1-3
Ctrl2
55 kDa
Cpx V
48 kDa
Cpx III
30 kDa
Cpx II
22 kDa
Cpx IV
18 kDa
Cpx I
Actine
VDAC1
